# Population-Based Screening or Targeted Screening Based on Initial Clinical Risk Assessment for Atrial Fibrillation: A Report from the Huawei Heart Study

**DOI:** 10.3390/jcm9051493

**Published:** 2020-05-15

**Authors:** Yutao Guo, Hao Wang, Hui Zhang, Yundai Chen, Gregory Y. H. Lip

**Affiliations:** 1 Department of Cardiology, Chinese PLA General Hospital, No.28, Fuxin Road, Beijing 100853, China; dor_guoyt@hotmail.com (Y.G.); wanghao_pla@126.com (H.W.); zhanghuiay08@sina.com (H.Z.); 2 Liverpool Centre for Cardiovascular Sciences, University of Liverpool, Liverpool, Merseyside L7 8TX, UK; 3 Aalborg Thrombosis Research Unit, Department of Clinical Medicine, Aalborg University, DK-9000 Aalborg, Denmark

**Keywords:** atrial fibrillation, screening, photoplethysmography, clinical risk score

## Abstract

Background: A general-population approach has been advocated to improve the screening of patients with atrial fibrillation (AF). A more pragmatic alternative may be targeted screening of patients at high risk of developing AF. We assess the value of a simple clinical risk score, C_2_HEST (C2, coronary artery disease/chronic obstructive pulmonary disease; COPD (1 point each); H, hypertension; E, elderly (age ≥75, doubled); S, systolic heart failure; HF (doubled); T, hyperthyroidism)); to facilitate population screening and detection of incident AF in the general population, in a prespecified ancillary analysis of the Huawei Heart Study. Methods: The Huawei Heart Study investigated general population screening for AF, identified using photoplethysmography (PPG)-based HUAWEI smart devices. We compared the value of a general population approach to a target screening approach between 26 October 2018 and 20 November 2019. Results: There were 644,124 individuals (mean age ± standard deviation, SD 34 ± 11; female 15.9%) who monitored their pulse rhythm using smart devices, among which 209,274 individuals (mean age 34 years, SD11; 10.6% female) completed the questionnaire on cardiovascular risk factors, with 739 detecting AF. Of these, 31.4% (*n* = 65,810) subjects reported palpitations. The median (interquartile range, IQR) duration to first detected AF was 11 (1–46), 6 (1–49), and 4 (1–24) in the population with low, intermediate, and high C_2_HEST score category, respectively (*p* = 0.03). Detected AF events rates increased with increasing C_2_HEST score points, stratified by age (*p* for trend, *p* < 0.001). Hazard ratios of the components of the C_2_HEST score for detected AF were between 1.31 and 2.75. A combination of symptomatic palpitations and C_2_HEST score increased prediction of AF detection, compared to using C_2_HEST score alone (c-indexes 0.72 vs. 0.76, Delong test, *p* < 0.001). Conclusions: The C_2_HEST score, especially when combined with symptoms, could facilitate a targeted population-based screening and preventive strategy for AF.

## 1. Clinical Perspective

### 1.1. What Is New

- A simple clinical risk score, C_2_HEST (C_2_, coronary artery disease/chronic obstructive pulmonary disease; COPD (1 point each); H, hypertension; E, elderly (age ≥75, doubled); S, systolic heart failure, HF (doubled); T, thyroid disease (hyperthyroidism)) facilitates population screening and detection of incident atrial fibrillation (AF) in the general population.
- The symptomatic C_2_HEST score, with the presence of associated palpitations, improves the predictive ability of detected AF, which might be useful for targeted screening.

### 1.2. What Are the Clinical Implications

- The general age ≥40 with >3 main cardiovascular risk factors (heart failure, hypertension, coronary artery disease, hyperthyroidism, diabetes, etc.) could be the candidates for AF screening.
- Using photoplethysmography (PPG)-based monitoring wristbands/wristwatches, with continuous frequent monitoring, there was a median of 4 days to the first detection of AF in the population with high-risk C_2_HEST score.

## 2. Introduction

Atrial fibrillation (AF) is the commonest heart rhythm disorder, which increases the risk of stroke, death, dementia, and heart failure. Most AF-related complications can be prevented with early diagnosis and appropriate intervention. However, early AF diagnosis and detection is problematic as patients are often asymptomatic, and the first presentation may be with an AF-related complication. While population screening has been advocated [1], new solutions with smart technologies have been developed to improve early detection [2,3,4,5]. 

The HUAWEI Heart Study investigated the effectiveness of AF screening in a large population-based cohort using smart device based photoplethysmography (PPG) technology [5]. It found that continuous home-monitoring with smart device-based PPG technology could be a feasible approach for AF screening, where the proportion of ‘suspected AF’ notifications was 0.2%, which increased with the highest proportion of ‘suspected AF’ detection of 2.8% in population aged ≥65 years [5]. This raises the question of whether AF screening should be a population-wide approach, with the associated logistic and cost issues, or should we use targeted screening of patients at high risk of developing AF?

Various clinical risk factors have been associated with incident AF [6], and several risk scores have been developed to predict incident AF, using commonly measured instrumental and laboratory variables [7,8,9]. Some more common and validated clinical factors have been used to derive a simple clinical risk score, (C_2_, coronary artery disease/chronic obstructive pulmonary disease; COPD (1 point each); H, hypertension; E, elderly (age ≥ 75, doubled); S, systolic heart failure, HF (doubled); T, thyroid disease (hyperthyroidism)) with good discrimination and internal calibration for incident AF [10]. The C_2_HEST score has been validated in retrospective analyses of a large healthy cohort and a post-stroke population [11,12]. The ability to identify high-risk subjects who could potentially be targeted for screening would allow for the optimization of healthcare resources and enhance the detection rates for AF, by focusing more intensive efforts for screening and detection of incident AF [12].

In this prespecified ancillary analysis of the HUAWEI Heart Study, our aim was to assess the value of the C_2_HEST score to facilitate population screening and detection of incident AF in a large prospective population-based screening study. Our principal objective was to investigate using the C_2_HEST score for a targeted population-based screening strategy. 

## 3. Methods

The design of Mobile health technology for improved screening, patient involvement, and optimizing integrated care in the Atrial Fibrillation (MAFA II) study program has been previously published [13]. Briefly, Huawei smart technology was used for screening of AF in the general population (referred to as the ‘Huawei Heart Study’), and the identified AF patients were then transferred into a structured program of holistic and integrated care using a smartphone App (mAF App) [13]. 

The adult population aged over 18 years old could freely use AF screening with smart devices based on PPG technology (Huawei Technologies Co., Ltd., Shenzhen, China) across China, once the participants have compatible Huawei smart device(s) and phone. These smart devices and phones included the Huawei phone (Android 5.0 or higher), and Huawei Watch GT (Version 1.0.3.52 or higher), Honor Watch (Version 1.0.3.52 or higher), and Honor Band 4 (Version 1.0.0.86 or higher). The predicting ability of the PPG algorithm (developed by Huawei) and smart devices have been validated before the massive general population screening study [14]. Subjects aged <18 years, and those with an inability to use a smart phone or devices were excluded. At least 14-day monitoring with smart devices based on PPG was proposed. The study was approved by the Central Medical Ethic Committee of Chinese PLA General Hospital (Approval number: S2017-105-02) and registered at www.chictr.org.cn (ChiCTR-OOC-17014138). The adult population downloading the AF screening App across China was enrolled into the present study between 26 October 2018 and 20 November 2019; this extends the data from our previous study report [5].

Study participants were required to fill in a questionnaire about palpitations and cardiovascular risk factors, using the AF screening App, before the study, then C_2_HEST scores were calculated automatically based on user-reported risk profiles. 

### 3.1. Definition of Main Cardiovascular Risk Factors

Hypertension was defined as a resting blood pressure ≥ 140 mm Hg systolic and/or ≥90 mm Hg diastolic on at least two occasions, or those having current antihypertensive drug treatment. CAD was defined as prior myocardial infarction, angina pectoris, percutaneous coronary intervention, or coronary artery bypass surgery. HF was defined as the presence of signs and symptoms of either right (elevated central venous pressure, hepatomegaly, dependent edema) or left ventricular failure (exertional dyspnea, cough, fatigue, orthopnoea, paroxysmal nocturnal dyspnoea, cardiac enlargement, rales, gallop rhythm, pulmonary venous congestion) or both, diagnosed by doctors. COPD is the chronic obstruction of lung airflow that interferes with normal breathing. Sleep apnea-hypopnea syndrome (OSAS) is apnea caused by upper airway obstruction during sleep, associated with frequent awakening and often with daytime sleepiness. Hyperthyroidism is the excessive functional activity of the thyroid gland with an excessive amount of thyroid hormones. Diabetes is defined as a fasting blood sugar level of 126 mg/dL (7 mmol/L) or higher, a random blood sugar level of more than 200 mg/dL (11.1 mmol/L) or higher. All of the above factors should be confirmed by doctors.

### 3.2. AF Detection and Confirmation

Once the participants downloaded the AF screening App, they freely decided to enter into the study after providing an electronic signature and authorization, periodic measurements would be automatically be taken every 10 min, and 60-s PPG signals would continuously be collected. The participants also could initiate active measurements at rest, and 45-s PPG signals would be collected [5]. As previously reported, the notification of ‘suspected AF’ using the PPG algorithm was sent to the participants, who were further confirmed by the health providers using the mAFA Telecare center and network hospitals, with clinical evaluation, electrocardiogram (ECG), or 24-h Holter [5]. 

### 3.3. Statistical Analysis

Baseline patient characteristics were summarized by reporting binary variables as proportions, and continuous variables by mean and standard deviation (SD). Data with a non-normal distribution were presented as median (interquartile range, IQR).

The clinical risk for AF was described according to levels of the C_2_HEST risk score for AF, defined as ‘low risk’ (C_2_HEST 0–1), ‘intermediate risk’ (C_2_HEST 2–3) and ‘high risk’ (C_2_HEST > 3). Time to first detected AF was calculated, categorized by C_2_HEST risk score. The Kruskal–Wallis H was utilized for the days to detected AF among C_2_HEST low, intermediate, and high risk strata.

The monitored AF event rates and risk in relation to C_2_HEST strata, among the population age 18–39, age 40–54, age 55–64, and age ≥65 were calculated to explore the ‘at risk’ population who could more possibly best benefit from general-population screening approach.

Using the Cox proportional hazards model, the association of individual components and detected AF was analyzed by calculating hazard rate ratios (HR, 95% confidential interval, CI). The receiver operating curves (ROC) and c-statistics were further calculated to assess the discrimination performance of the score. We also investigated using the C_2_HEST plus (symptomatic) palpitation, and the diagnostic accuracy of this modified approach (symptoms plus C_2_HEST score, i.e., a ‘symptomatic C_2_HEST score’) was compared to C_2_HEST score alone, for AF detection, with c-indexes compared using the DeLong equality test

A two-sided *p*-value < 0.05 was considered as statistically significant. Statistical analysis was performed using IBM SPSS Statistics, version 25.0 (SPSS Inc., Chicago, IL, USA), and MedCalc 12.6.1.0 (MedCalc Software Ltd, Ostend, Belgium).

## 4. Results

Between 26 October 2018 and 20 November 2019, there were 644,124 individuals (mean age 34 years, standard deviation, SD 11; female 15.9%) who monitored their pulse rhythm using smart devices, among which 209,274 individuals (mean age 34 years, SD 11; 10.6% female) completed the questionnaire on cardiovascular risk factors. Mean C_2_HEST was 1.2 (SD 1.1) in those aged ≥55 years, and 31.4% (65,810) reported palpitations. Baseline characteristics of the 209,274 subjects are summarised in Table 1. The median duration to first detected AF was 4 days (interquartile range, IQR 1–24) in population with a ‘high risk’ C_2_HEST score category, which was shorter compared to intermediate and low risk C_2_HEST scores (*p* = 0.03) (Table 2). There were 739 individuals who received the notification of detected AF by the PPG algorithm (Supplementary Figure S1).

Detected AF events rates increased with increasing C_2_HEST score points, or with risk strata (low, intermediate, and high risk), in both the general population and population with palpitations (all *p* for trend, <0.001, Table 3). Detection of AF also increased with increasing C_2_HEST, even when stratified by age 18–39, age 40–54, age 55–64, and age ≥65 (Table 4). The rates of detected AF among subjects with high-risk C_2_HEST score at age 18–39, age 40–54, age 55–64, and age ≥65 were 0.46%, 3.57%, 6.57%, and 10.28%, respectively (Table 4A). Individuals aged >40 with C_2_HEST scores >3 and aged ≥55 with C_2_HEST scores >3 had detected AF rates of >3% (Table 4A, red figures). 

**Figure 1:**
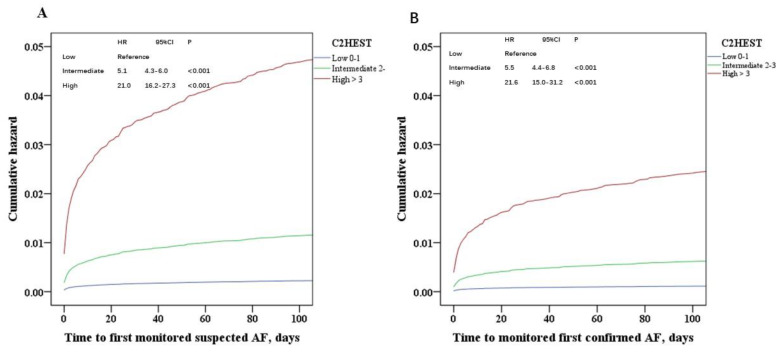
The cumulative risk of monitored AF, in relation to C_2_HEST. (**A**) Suspected AF, (**B**) Confirmed AF. HR, hazard ratio. CI, confidential interval.

On multivariable analysis, components of the C_2_HEST score contributed to the risk for detected AF, with Hazard Ratios (HRs) between 1.31 and 2.75. HF was the greatest factor for confirmed AF (HR 2.75; 95% CI 2.17–3.48) (Table 5). Cumulative risks of monitored AF, in relation to C_2_HEST score are shown in Figure 1.

**Figure 2:**
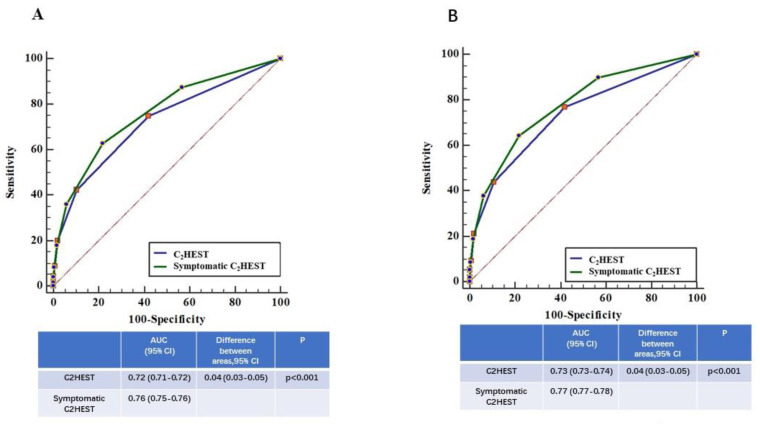
Comparison of ROC curves of C_2_HEST and symptomatic C_2_HEST. (**A**) Suspected AF, (**B**) Confirmed AF. Symptomatic C_2_HEST: C_2_HEST plus palpitation. AUC, areas under the curve. CI, confidential interval.

The C indexes (95% CI) were 0.72 (0.71–0.72) and 0.76 (0.75–0.76) for C_2_HEST score without and with symptoms (palpitations, i.e., symptomatic C_2_HEST score), respectively (Figure 2). The symptomatic C_2_HEST score increased the predictive ability for detected AF compared to using the C_2_HEST score alone, with a difference between area under curve areas (95% confidential interval) of 0.04 (95% CI 0.03–0.05) (Delong test, all *p* < 0.05).

## 5. Discussion

In this large prospective population screening study, our main findings are as follows: (i) an increasing increased C_2_HEST score was associated with a decreased time to detected AF, with a median of 4 days to first detection; (ii) the risk for detected AF in population with C_2_HEST > 3 was increased compared to those with low C_2_HEST scores, with greater detection seen in those aged > 40 with C_2_HEST scores > 3 and aged ≥ 55 with C_2_HEST scores > 2; and (iii) a high C_2_HEST score in a symptomatic patient was associated with a greater predictive ability for detected AF.

The C_2_HEST score was first derived from a large cohort of 471,446 Chinese patients and was externally validated in the Korean National Health Insurance Service Health Screening cohort with 514,764 Korean patients [10]. In large hospital-based and community-based cohorts, the C_2_HEST score has been validated with good performance in predicting AF among healthy and high-risk populations [11,12]. The present large prospective population study further extends the validation of the C_2_HEST score for the predictive ability of detected AF with PPG screening technology in a prespecified analysis of the Huawei Heart Study. 

The time to first detected AF varies with screening technology and devices [1], as well as the population’s risk profile. In a high-risk population, for example, two weeks of twice-daily intermittent AF screening may be warranted using single-point handheld monitoring [1,15]. Nevertheless, continuous and more intensive monitoring using smart technology would improve AF detection, with a median of 4 days in the present study. The positive predictive value (PPG) of detecting AF was 91.6% in the Huawei Heart Study with periodic measurements every 10 min in the baseline, compared to 71.3% in the Apple Heart Study with periodic measurements every 2 h [4,5]. 

The use of smart technology with wristbands or wristwatches could possibly be more comfortable and acceptable than e-patches for long monitoring periods. For example, approximately one-third of subjects in an e-patch study never wore the e-patch, and 40 subjects reported skin irritation and stopped monitoring [2], which has not been reported with PPG-based smartwear [4,5]. 

Although the risk for detected AF was increased among high-risk C_2_HEST scores, there were different detected AF events rates in relation to age strata. For example, the rates of detected AF were 0.46%, 3.57%, 6.57%, and 10.28%, respectively, among the screened high-risk C_2_HEST score population at different age strata, i.e., 18–39, 40–54, 55–64, and ≥66, respectively. Indeed, age is an independent risk factor for AF and its related complications [16]. When other clinical risk factors occurred, such as HF, hypertension, etc—the ’young’ population would be at increased risk for AF occurrence. Indeed, those aged >40 with C_2_HEST scores > 3 and aged ≥55 with C_2_HEST scores >2 had detected AF rates of over 3%. Thus, the general population who are aged ≥40 with >3 main cardiovascular risk factors (hypertension, OSAS, HF, Diabetes, etc.) could be candidates for AF screening.

Clinical epidemiological data have shown that common clinical factors such as HF, hypertension, CAD, hyperthyroidism, and COPD/OSAS are clearly associated with the risk for incident AF [17,18,19]. Nevertheless, the risks for AF might be different between Western and Chinese populations. Indeed, we show that the greatest risk at all age cohorts for detected AF was with HF, followed by hypertension, hyperthyroidism, and diabetes in the Chinese population, while in the Danish population, COPD was the greatest risk for new-onset AF [12]. 

The clinical scores which would contribute to improving screening for AF should be weighted for the components of the more common (and validated) risk factors related to incident AF. Some indices, such as PR interval, echocardiographic measurements, clinically significant cardiac murmur, would be much less available in a ’healthy’ population [7], besides, clinical risk factors per se generally have only moderate predictive ability for incident AF (c-indexes approx. 0.6–0.7) [9]. As a simple clinical tool, the C_2_HEST score could identify the population who most likely are at the high risk for AF occurrence, were combined with the smart-technology and continuous monitoring, may be helpful for improving early AF detection. 

Of note, our study also showed that a symptomatic C_2_HEST score with the presence of associated palpitations improved the predictive ability of detected AF, which might be useful for targeted screening, especially if resources are more limited. Thus, a middle-aged adult with symptoms and 2 clinical risk factors may benefit from more intensive AF screening. An ongoing formal health economic analysis would assess the cost-effectiveness of such an approach, compared to general population screening.

### Limitation

The main limitations of the present study were that this was a ‘young’ population and there was self-reporting of AF risk factors. There were 32% of individuals who completed the electronic questionnaire on cardiovascular risk factors, which was used for the calculation of C_2_HEST score. Given that this was a prospective large population-based screening study, it would be difficult to collect data individually. There was less females in this study, which may reflect the males may like to use the smart electronic devices. The implication for females would need more validation. Another limitation was that the rate of follow-up was 62%, although 88% (*n =* 374) were confirmed AF with the medical history, physical examination, ECG, 24-h Holter, etc. The relative low follow-up could lower the event rate of confirmed AF. However, a sensitivity analysis on both suspected AF and confirmed AF were consistent with the main finding. Finally, it was not our objective to compare against other clinical scores for AF prediction, as our objective was to focus on using the C_2_HEST score for a targeted population-based screening strategy. Indeed, the present study is not asking the question whether the use of the C_2_HEST score improved clinical outcomes, and the C_2_HEST score has already been previously compared against the CHADS_2_, CHA_2_DS_2_VASc, and Framingham scores in prior studies [10,11,12]. However, there would need more evidence in the application of C_2_HEST facilitated target population-based screening strategy in European or other populations. 

## 6. Conclusions

In this prespecified ancillary analysis of the HUAWEI Heart Study, we show that the C_2_HEST score, especially when combined with symptoms, could facilitate a targeted population-based screening and preventive strategy for AF.

## Figures and Tables

**Table 1:** Baseline characteristics of 209,274 subjects with C_2_HEST score.

Index	Age 18–39 (*n =* 145,389)	Age 40–54 (*n =* 52,089)	Age 55–64 (*n =* 8716)	Age ≥ 65 (*n =* 3080)
Female	15,339 (10.6%)	4556 (8.7%)	1423 (16.3%)	875 (28.4%)
C_2_HEST mean (SD)	0.43 (0.37)	0.78 (0.68)	1.07 (0.94)	1.72 (1.53)
0	94,605 (65.1%)	22,837 (43.8%)	2902 (33.3%)	677 (22.0%)
1	41,772 (28.7%)	20,075 (38.5%)	3252 (37.3%)	859 (27.9%)
2	7548 (5.2%)	7745 (14.9%)	1895 (21.7%)	765 (24.8%)
3	1031 (0.7%)	1040 (2.0%)	469 (5.4%)	419 (13.6%)
4	235 (0.2%)	225 (0.4%)	123 (1.4%)	198 (6.4%)
5	62 (0.0%)	119 (0.2%)	66 (0.8%)	109 (3.5%)
6+	136 (0.1%)	48 (0.1%)	9 (0.1%)	53 (1.7%)
Palpitation	43,018 (29.6%)	18,093 (34.7%)	3297 (37.8%)	1402 (45.5%)
Hypertension	13,318 (9.2%)	13,587 (26.1%)	3552 (40.8%)	1530 (49.7%)
COPD/OSAS	42,022 (28.9%)	21,732 (41.7%)	3445 (39.5%)	1156 (37.5%)
CAD	1047 (0.7%)	2630 (5.0%)	1502 (17.2%)	967 (31.4%)
Diabetes	2088 (1.4%)	3538 (6.8%)	1408 (16.2%)	586 (19.0%)
Heart failure	1950 (1.3%)	805 (0.4%)	304 (0.1%)	227 (0.1%)
Hyperthyroidism	1740 (1.2%)	909 (1.7%)	218 (2.5%)	75 (2.4%)

Reported as N (%) unless specified otherwise. SD, standard deviation. COPD, chronic obstructive pulmonary disease. OSAS, sleep apnea-hypopnea syndrome. CAD, coronary artery disease.

**Table 2:** Time to monitored first detected AF (days), in relation to C_2_HEST.

Risk Score	Detected AF (*n =* 739)
C_2_HEST	
0	11 (1–46)
1	11 (2–46)
2	6 (1–38)
3	7 (2–64)
4	5 (1–31)
5	3 (1–29)
6+	5 (0–19)
Low 0–1	11 (1–46)
Intermediate 2–3	6 (1–49)
High > 3	4 (1–24)
*p*	0.03

Reported as median (interquartile range). The days to first suspected AF was tested with Kruskal–Wallis H among C_2_HEST low, intermediate, and high level.

**Table 3:** Detected AF, in relation to C_2_HEST distribution, in the general population and those with palpitations.

Risk Score	General Population (*n* = 209,274)			Population with Palpitations (*n* = 65,810)		
C_2_HEST	Detected AF, *n*	Total Number, *n*	Detected Rate of AF, %	Detected AF, *n*	Total Number, n	Detected Rate of AF, %
0	186	121,021	0.15%	93	30,487	0.31%
1	241	65,958	0.37%	153	23,603	0.65%
2	165	17,953	0.92%	119	8468	1.41%
3	81	2959	2.74%	66	2086	3.16%
4	34	781	4.35%	29	613	4.73%
5	20	356	5.62%	19	317	5.99%
6+	12	246	4.88%	12	236	5.08%
Low 0–1	427	186,979	0.23%	246	54,090	0.45%
Intermediate 2–3	246	20,912	1.18%	185	10,554	1.75%
High > 3	66	1383	4.77%	60	1166	5.15%
*p* for trend			<0.001			<0.001

**Table 4:** Monitored AF event rates, in relation to C_2_HEST score and risk strata, among population age 18–39, age 40–54, age 55–64 and age ≥65. (**A**) Suspected AF. (**B**) Confirmed AF.

(A) Suspected AF.																
Detected AF (*n =* 739)	Age 18–39 (*n =* 145,389)				Age 40–54 (*n =* 52,089)				Age 55–64 (*n =* 8716)				Age ≥ 65 (*n =* 3080)			
C_2_HEST	AF	Number, *n*	Rate, %	HR (95%CI)	AF	Number, *n*	Rate, %	HR (95%CI)	AF	Number, *n*	Rate, %	HR (95%CI)	AF	Number, *n*	Rate,%	HR (95% CI)
0	55	94,605	0.06%	1.00 (reference)	82	22,837	0.36%	1.00 (reference)	28	2902	0.96%	1.00 (reference)	21	677	3.10%	1.00 (reference)
1	39	41,772	0.09%	1.59 (1.06–2.40)	107	20,075	0.53%	1.48 (1.11–1.98)	58	3252	1.78%	1.83 (1.17–2.88)	37	859	4.31%	1.38 (0.80–2.36)
2	19	7548	0.25%	4.29 (2.54–7.23)	55	7745	0.71%	1.98 (1.41–2.79)	51	1895	2.69%	2.79 (1.76–4.43)	40	765	5.23%	1.64 (0.97–2.79)
3	6	1031	0.58%	10.18 (4.38–23.64)	18	1040	1.73%	4.90 (2.94–8.17)	25	469	5.33%	5.58 (3.25–9.57)	32	419	7.64%	2.48 (1.42–4.30)
4	1	235	0.43%	7.54 (1.04–54.46)	8	225	3.56%	10.03 (4.85–20.73)	10	123	8.13%	8.66 (4.21–17.84)	15	198	7.58%	2.34 (1.20–4.55)
5	0	62	0.00%	-	4	119	3.36%	9.38 (3.44–25.60)	2	66	3.03%	3.19 (0.76–13.39)	14	109	12.84%	4.69 (2.38–9.22)
6+	1	136	0.74%	13.18 (1.82–95.29)	2	48	4.17%	11.94 (2.93–48.57)	1	9	11.11%	11.21 (1.52–82.43)	8	53	15.09%	5.06 (2.24–11.44)
Low 0–1	94	136,377	0.07%	1.00 (reference)	189	42912	0.44%	1.00 (reference)	86	6154	1.40%	1.00 (reference)	58	1536	**3.78%**	**1.00 (reference)**
Intermediate 2–3	25	8579	0.29%	4.21 (2.71–6.54)	73	8785	0.83%	1.89 (1.44–2.48)	76	2364	**3.21%**	**2.31 (1.70–3.15)**	72	1184	**6.08%**	**1.59 (1.13–2.26)**
High > 3	2	433	0.46%	6.86 (1.69–27.83)	14	392	**3.57%**	**8.19 (4.76–14.10)**	13	198	**6.57%**	**4.81 (2.68–8.63)**	37	360	**10.28%**	**2.78 (1.84–4.21)**
*p* for trend			<0.001				<0.001				<0.001				<0.001	
**(B) Confirmed AF.**																
**Confirmed AF (*n =* 374)**	**Age 18–39 (*n =* 145,389)**				**Age 40–54 (*n =* 52,089)**				**Age 55–64 (*n =* 8716)**				**Age ≥ 65 (*n =* 3080)**			
**C_2_HEST**	**AF**	**Number, *n***	**Rate, %**	**HR (95%CI)**	**AF**	**Number, *n***	**Rate, %**	**HR (95% CI)**	**AF**	**Number, *n***	**Rate,%**	**HR (95%CI)**	**AF**	**Number, *n***	**Rate, %**	**HR (95% CI)**
0	22	94,605	0.02%	1.00 (reference)	41	22,837	0.18%	1.00 (reference)	16	2902	0.55%	1.00 (reference)	7	677	1.03%	1.00 (reference)
1	17	41,772	0.04%	1.74 (0.92–3.28)	53	20,075	0.26%	1.47 (0.98–2.21)	31	3252	0.95%	1.72 (0.94–3.14)	23	859	2.68%	2.58 (1.10–6.02)
2	8	7548	0.11%	4.52 (2.01–10.15)	22	20,075	0.11%	1.59 (0.94–2.66)	31	1895	1.64%	2.97 (1.62–5.43)	24	765	3.14%	2.93 (1.26–6.81)
3	2	1031	0.19%	8.43 (1.98–35.87)	11	1040	1.06%	5.99 (3.08–11.65)	15	469	3.20%	5.88 (2.90–11.89)	17	419	4.06%	3.89 (1.61–9.40)
4	0	235	0.00%	-	2	225	0.89%	5.03 (1.21–20.79)	7	123	5.69%	10.56 (4.34–25.68)	5	198	2.53%	2.26 (0.71–7.17)
5	0	62	0.00%	-	3	119	2.52%	14.08 (4.36–45.49)	2	66	3.03%	5.54 (1.27–24.11)	8	109	7.34%	8.23 (2.98–22.72)
6+	0	136	0.00%	-	1	48	2.08%	11.90(1.63–86.52)	1	9	11.11%	19.45 (2.58–146.73)	5	53	9.43%	9.47 (3.00–29.84)
Low 0–1	39	136,377	0.03%	1.00 (reference)	94	42,912	0.22%	1.00 (reference)	47	6154	0.76%	1.00 (reference)	30	1536	1.95%	1.00 (reference)
Intermediate 2–3	10	8579	0.12%	4.06 (2.02–8.12)	33	8785	0.38%	1.72 (1.15–2.56)	46	2364	1.95%	2.56 (1.70–3.85)	41	1184	3.46%	1.74 (1.08–2.79)
High > 3	0	433	0.00%	-	6	392	1.53%	7.07 (3.09–16.14)	10	198	5.05%	6.73 (3.40–13.33)	18	360	5.00%	2.59 (1.44–4.67)
*p* for trend			<0.001					<0.001				<0.001			<0.001	

HR, hazard ratio. CI, confidential interval.

**Table 5:** Univariable and multivariable hazard ratios for detected AF in 209,274 subjects.

Detected AF (*n =* 739)	Univariable		Multivariable	
	HR (95% CI)	*p*	HR (95% CI)	*p*
Male	1.29(1.04–1.61)	0.021	1.69 (1.35–2.11)	<0.001
Age	1.10 (1.09–1.11)	<0.001	1.09 (1.08–1.10)	<0.001
Palpitation	4.32(3.71–5.04)	<0.001	3.07 (2.61–3.61)	<0.001
Heart failure	10.52 (8.55–12.95)	<0.001	2.75 (2.17–3.48)	<0.001
CAD	9.80 (8.25–11.64)	<0.001	1.15 (0.93–1.41)	0.18
Hypertension	3.64 (3.14–4.21)	<0.001	1.16 (0.99–1.36)	0.06
Hyperthyroidism	3.29 (2.32–4.67)	<0.001	1.47 (1.02–2.11)	0.034
Diabetes	3.18 (2.52–4.02)	<0.001	1.31 (1.03–1.69)	0.029
COPD/OSAS	1.55 (1.34–1.80)	<0.001	0.95 (0.82–1.11)	0.58
**Confirmed AF (*n =* 374)**	**Univariable**		**Multivariable**	
Male	0.76 (0.56–1.02)	0.07	1.75 (1.28–2.38)	<0.001
Age	1.11(1.10–1.12)	<0.001	1.10 (1.09–1.11)	<0.001
Palpitation	4.61 (3.71–5.73)	<0.001	3.25 (2.58–4.09)	<0.001
Heart failure	10.15 (7.55–13.64)	<0.001	2.37 (1.69–3.31)	<0.001
CAD	9.85 (7.74–12.55)	<0.001	1.03 (0.77–1.37)	0.82
Hypertension	4.21 (3.43–5.17)	<0.001	1.29 (1.03–1.61)	0.024
Hyperthyroidism	3.99 (2.54–6.27)	<0.001	1.79 (1.12–2.85)	0.014
Diabetes	3.19 (2.30–4.43)	<0.001	1.40 (1.00–2.00)	0.053
COPD/OSAS	1.67 (1.36–2.04)	<0.001	1.02(0.82–1.25)	0.87

HR: hazard ratio. CAD, coronary artery disease. COPD, chronic obstructive pulmonary disease. OSAS, sleep apnea-hypopnea syndrome.

## Supplementary Materials

The following are available online at https://www.mdpi.com/2077-0383/9/5/1493/s1, Figure S1: Screening and confirmation of AF.
