# Supplementary figures and images for: Population-Based Screening or Targeted Screening Based on Initial Clinical Risk Assessment for Atrial Fibrillation: A Report from the Huawei Heart Study
Source: J Clin Med. 2020 May 15;9(5):1493. doi: 10.3390/jcm9051493 (PMC7291296; doi:10.3390/jcm9051493)

## Supplementary

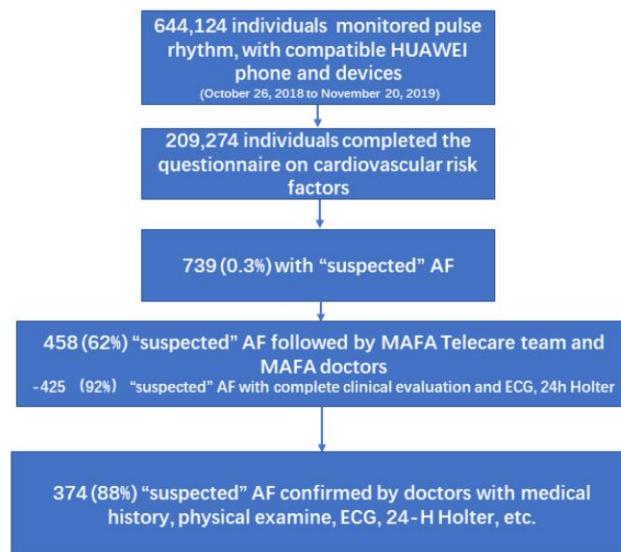

**Figure S1.** Screening and confirmation of AF.

Supplement: Supplementary file 1 [file jcm-09-01493-s001.pdf]
